# Simultaneous GA and CNV/MNV: incidence, characteristics, and treatments

**DOI:** 10.1007/s00417-024-06721-5

**Published:** 2025-04-14

**Authors:** Keiko Kataoka, Richard Gale, Xiaoxin Li, Figen Şermet, Cynthia X. Qian, Chui Ming Gemmy Cheung, Miltiadis K. Tsilimbaris, Igor Kozak

**Affiliations:** 1 Kyorin University School of Medicine, Tokyo, Japan; 2 Hull York Medical School, University of York and York Teaching Hospital NHS Foundation Trust, York, UK; 3 Eye Center and Eye Institute of Peking University People’s Hospital, Beijing, China; 4 Ophthalmology Department, Ankara University School of Medicine, Ankara, Turkey; 5 Department of Ophthalmology, University of Montreal, Montreal, Canada; 6 Singapore Eye Research Institute, National Eye Centre, Singapore, Singapore; 7 Duke-NUS Medical School, National University of Singapore, Singapore, Singapore; 8 University of Crete Medical School, Crete, Greece; 9 Moorfields Eye Hospital Centre, Abu Dhabi, United Arab Emirates; 10 University of Arizona-Tucson, Tucson, AZ USA

**Keywords:** Age-related macular degeneration, Choroidal neovascularization, Macular neovascularization, Geographic atrophy

## Abstract

**Purpose:**

Understanding the clinical characteristics and underlying mechanisms of simultaneous geographic atrophy (GA) and choroidal neovascularization (CNV)/macular neovascularization (MNV) is necessary for the long-term management of late age-related macular degeneration (AMD) in clinical practice.

**Methods:**

The authors reviewed the literature on the incidence, risk factors, and clinical characteristics of simultaneous GA and CNV/MNV and developed consensus recommendations for the diagnosis, assessment, and management of simultaneous GA and CNV/MNV in clinical practice.

**Results:**

The incidence rate of CNV/MNV in eyes with GA is reported as 7.4% per patient-year or 13.8% in 4.1 years, while that of macular atrophy (MA) subsequent to CNV/MNV is reported as 24.4% to 37% in 24 months. Recent studies using optical coherence tomography angiography (OCT-A) revealed the presence of subclinical CNV/MNV in 11% to 16% of eyes with GA. Fundus autofluorescence is used to detect MA; optical coherence tomography (OCT) and OCT-A are useful for detecting MA, especially around the fovea, with OCT-A offering high sensitivity and specificity in the detection of both MA and CNV/MNV. GA and CNV/MNV share the genetic risk factors of *HTRA1*, complement factor H, complement factors 3 and 2, and *ARMS2*, and clinical risk factors of large drusen, cuticular drusen, intraretinal hyperreflective foci, and subretinal drusenoid deposits, suggesting that simultaneous GA and CNV/MNV represents a continuum of AMD. Anti–vascular endothelial growth factor therapy for CNV/MNV is reported to have no impact on the speed or magnitude of MA development or enlargement. An association has been observed between CNV subtype and MA progression, with the latter being slower in the presence of type 1 CNV/MNV.

**Conclusions:**

These findings suggest there is a high probability of coexistence of GA and CNV/MNV and that they should not be considered separately. Future clinical studies should assess the two conditions simultaneously using OCT and OCT-A.

**Key Messages:**

***What is known***

- Owing to differences in their clinical characteristics, geographic atrophy (GA) and choroidal neovascularization (CNV)/macular neovascularization (MNV) have historically been regarded as two separate entities; however, several cases of coexistent GA and CNV/MNV have been reported recently in the published literature.

***What is new***

- The findings of this review confirm that GA and CNV/MNV share common genetic risk factors and clinical characteristics, and suggest that these two entities are part of a continuum of late-stage age-related macular degeneration (AMD).
- The potential for GA and CNV/MNV to coexist should be considered in any discussion of the long-term management of late AMD; moreover, clinicians should assess for CNV/MNV in patients with GA, and for GA in those with CNV/MNV, using multimodal imaging.

**Supplementary Information:**

The online version contains supplementary material available at 10.1007/s00417-024-06721-5.

## Introduction

Macular neovascularization (MNV) and geographic atrophy (GA) can occur in the late stages of age-related macular degeneration (AMD), which is a leading cause of blindness in the elderly worldwide [1]. MNV is newly formed pathological neovascularization from the choriocapillaris into the sub–retinal pigment epithelium (RPE) space (type 1 MNV) or subretinal space (type 2 MNV), or from the retinal deep capillary plexus (type 3 MNV) [2]. GA, or macular atrophy, is characterized by a sharply defined round or oval area of hypopigmentation or depigmentation with increased visibility of the underlying choroidal vessels due to the loss of RPE and thinning or loss of the outer retina [3]. The term “GA” has been used to describe macular atrophy in the absence of MNV, whereas the term “macular atrophy” is used regardless of the presence or absence of MNV [3]. In the past, GA and MNV were considered two different entities because of their different clinical characteristics. However, there have been several reports of coexistent GA and choroidal neovascularization (CNV)/MNV [4–6]. In general, clinical studies have focused on only CNV/MNV or GA individually, and treatment options for simultaneous GA and CNV/MNV are currently lacking.

Therefore, understanding the underlying mechanisms and the clinical characteristics of simultaneous GA and CNV/MNV is necessary for the management of late AMD. This review aims to discuss the incidence, risk factors, and clinical characteristics of simultaneous GA and CNV/MNV. In addition, it provides guidance for the diagnosis and management of simultaneous GA and CNV/MNV in clinical practice.

## Methods

This article is based on a review of the literature and a consensus among retinal experts from the Vision Academy. The Vision Academy is a group of over 80 international experts who, through their collective expertise, provide consensus guidance for managing clinically challenging situations, especially in areas of controversy or with insufficient conclusive evidence (www.visionacademy.org). The Vision Academy is sponsored by Bayer.

A comprehensive literature search was performed using the PubMed database (cut-off date: July 11, 2022). The keywords for the literature search included “simultaneous” or its synonyms, “geographic atrophy” or its synonyms, and “choroidal neovascularization” or its synonyms. The detailed search strategy is shown in Supplementary Table 1. After screening, a total of 40 studies were selected for inclusion in the review (Supplementary Fig. 1). These 40 key studies were critically reviewed and are summarized in Tables 1, 2, 3, 4 and 5.

**Table 1:** Studies of simultaneous GA and MNV/CNV

Citation	Study design	Indication(s)	*n*	Length of follow-up	Key findings
Grob et al. (2012) [37]	Prospective, observational, single-center study	Simultaneous GA and CNV/CNV alone/GA alone	328 patients	NA	In eyes with simultaneous GA and CNV, GA often preceded CNV. Allelic frequency of *HTRA1*, *CFH*, *C3*, and *C2* did not differ in patients with simultaneous GA and CNV vs. GA or CNV alone
Saade et al. (2014) [32]	Prospective, observational, single-center study	Late AMD	58 patients	NA	Patients with simultaneous GA and CNV were older (86.4 vs. 81.8 years; *p* = 0.049) and had higher prevalence of advanced AMD in the fellow eyes (81.3% vs. 31.0%; *p* < 0.001) than patients with GA or CNV alone. Frequency of *CFH* and *ARMS2* risk alleles did not differ in simultaneous GA and CNV vs. GA or CNV alone
Chakravarthy et al. (2018) [4]	Retrospective, observational, multicenter study	Bilateral GA	1901 patients	Median 1.4 years (IQR, 0.6–2.8)	Rate of CNV progression in eyes with GA was 7.4% per patient-year
Hwang et al. (2021) [5]	Post hoc analysis of a multicenter study	GA	456 eyes	Mean 4.1 years (SD, 1.4 years)	13.8% of eyes with GA demonstrated exudative nAMD. GA enlargement rate was similar between GA with subsequent exudative nAMD and without subsequent exudative nAMD
de Oliveira Dias et al. (2018) [21]	Prospective, observational, single-center study	GA in one eye/CNV in fellow eye	50 eyes	12 months	16.0% of eyes with GA had subclinical CNV/MNV
Capuano et al. (2017) [6]	Retrospective, observational, multicenter study	GA	644 eyes	Mean 45.7 months (SD, 14.7 months)*	Quiescent CNV was found in 11% of eyes with GA. Of 19 eyes selected for analysis, 26% developed exudation during the follow-up period. The area of quiescent CNV was spared from atrophy

*The stated follow-up period applies specifically to the 19 eyes selected for analysis

*AMD*, age-related macular degeneration; *ARMS2*, age-related maculopathy susceptibility 2; *C2*, complement factor 2; *C3*, complement factor 3; *CFH*, complement factor H; *CNV*, choroidal neovascularization; *GA*, geographic atrophy; *HTRA1*, high-temperature requirement factor A1; *IQR*, interquartile range; *MNV*, macular neovascularization; *NA*, not applicable; *nAMD*, neovascular age-related macular degeneration; *SD*, standard deviation

**Table 2:** Studies of macular atrophy subsequent to MNV/CNV

Citation	Study design/publication type	Indication	*n*	Age, years, mean	Treatment	Length of follow-up	Key findings
Cohen et al. (2012) [40]	Retrospective, observational, multicenter study	nAMD	290 eyes	NA	Ranibizumab	12 months	Overall, 7.2% of the eyes studied lost ≥ 15 letters due to the progression of macular atrophy, fibrosis, subretinal hemorrhage, or RPE tear
Rofagha et al. (2013) [9]	Post hoc analysis of a multicenter study	nAMD	65 patients	74.9 (SD 7.7)	Ranibizumab	Mean 7.3 years (SD, 0.46 years)	Macular atrophy was detected in 98% of eyes and was significantly correlated with poor visual outcome at 7 years
Ying et al. (2015) [41]	Post hoc analysis of a multicenter study	nAMD	1014 patients	Number of patients: 50–69 years: 127 70–79 years: 357 80–89 years: 465 ≥ 90 years: 65	Ranibizumab/bevacizumab, monthly/PRN	2 years	Eyes with GA at baseline had less VA gain at year 2 than eyes without GA (*p* = 0.04)
Sadda et al. (2018) [10]	Post hoc analysis of a multicenter study	nAMD	1095 patients	0.5 mg/monthly: 78.8 (SD 8.4) 0.5 mg/PRN: 78.5 (SD 8.3) 2.0 mg/monthly: 79.3 (SD 8.3) 2.0 mg/PRN: 78.3 (SD 8.3)	Ranibizumab 0.5 mg/2.0 mg monthly/PRN	24 months	Baseline presence of intraretinal cyst, GA in fellow eyes, and increase in age were associated with an increased incidence of macular atrophy, whereas baseline subretinal fluid was associated with a decreased risk of macular atrophy development. Monthly vs. PRN anti-VEGF treatment trended toward an association with macular atrophy
Daniel et al. (2018) [17]	Post hoc analysis of a multicenter study	nAMD with fibrotic scar	68 eyes	NA	Ranibizumab/bevacizumab, monthly/PRN	5 years	Macular atrophy developed in 54% of eyes with fibrotic scarring, was positioned within or adjacent to the scar, and could encircle the scar either partially or completely
Amaro et al. (2012) [39]	Retrospective, observational, photography database center study	nAMD with pre-existing GA	11 eyes	NA	Ranibizumab/ bevacizumab	Mean 20 months (SD, 10 months)	Anti-VEGF therapy was effective for the treatment of simultaneous GA and CNV
Bhisitkul et al. (2016) [18]	Post hoc analysis of a multicenter study	nAMD	48 patients	NA	Ranibizumab	7 years	High frequency ranibizumab therapy improved long-term vision in study eyes vs. untreated fellow eyes and was not associated with macular atrophy
Arevalo et al. (2016) [14]	Retrospective, observational, multicenter study	nAMD	292 eyes	74.6 (SD 8.1)	Bevacizumab	5 years	Macular atrophy was present in 16% of eyes at baseline, increasing to 42.5% of eyes at 5 years. The number of anti-VEGF injections did not differ between eyes with vs. eyes without macular atrophy
Bailey et al. (2019) [12]	Post hoc analysis of a multicenter study	nAMD	596 eyes	NA	Ranibizumab/bevacizumab, monthly/PRN	24 months	Intralesional macular atrophy was present in 9.6% of eyes at baseline. Incident intralesional macular atrophy occurred in 24.4% of eyes and extralesional macular atrophy in only 1.54% of eyes by 24 months. Intralesional macular atrophy at the final visit was lower in eyes with predominantly classic CNV at baseline, subretinal fluid at final visit, or pigment epithelial detachment at final visit. No significant association with incidence or progression of intralesional macular atrophy was observed in patients treated with ranibizumab vs. bevacizumab and monthly vs. PRN regimens
Gune et al. (2020) [11]	Post hoc analysis of a multicenter study	nAMD	761 patients	77.9 (SD 8.26)	Ranibizumab 0.5 mg/2.0 mg monthly/PRN	24 months	Rates of new macular atrophy development and macular atrophy enlargement were similar with ranibizumab 0.5 mg vs. 2.0 mg and monthly vs. PRN regimen
Gillies et al. (2020) [13]	Post hoc analysis of a multicenter study	nAMD	278 patients	Ranibizumab: 76.6 (SD 8.5) Aflibercept: 78.7 (SD 7.5)	Ranibizumab/aflibercept, T&E	24 months	No significant differences in the rate of development or growth of macular atrophy over 24 months were observed in nAMD patients treated with ranibizumab vs. aflibercept using an identical T&E regimen
Siedlecki et al. (2021) [19]	Retrospective, observational, single-center study	GA with subsequent CNV	6 patients	76.8 (SD 2.0)	Ranibizumab/aflibercept/bevacizumab	1.42 ± 0.48 years before and 3.64 ± 2.73 years after CNV	No significant difference was observed in mean GA growth rate before vs. after anti-VEGF therapy
Sadda et al. (2020) [44]	Post hoc analysis of a multicenter study	nAMD	761 patients	77.9 (SD 8.26)	Ranibizumab 0.5 mg/2.0 mg monthly/PRN	24 months	OCT analyses identified the following baseline risk factors for macular atrophy development: increased central drusen volume, reduced choroidal thickness, presence of nascent atrophy, reticular pseudo drusen, increased central foveal thickness, intraretinal cysts, type 3 neovascularization, atrophy in the fellow eye, and the absence of subretinal fluid
Daniel et al. (2016) [20]	Post hoc analysis of a multicenter study	RAP	1183 eyes	With RAP: 81.7 (SE 0.65) Without RAP: 79.0 (SE 0.23)	Ranibizumab/bevacizumab, monthly/PRN	2 years	Macular atrophy was more common in eyes with RAP (31.8%) compared with those without RAP (19.4%; *p* = 0.004)
Tanaka et al. (2015) [15]	Retrospective, observational, single-center study	nAMD	81 eyes	Median (IQR), 78 (72–83)	Ranibizumab/bevacizumab	Mean 4.9 years (range, 3.6–6.6 years)	Macular atrophy within or overlapping the boundary of the CNV lesion was present in 5% of eyes and macular atrophy outside the CNV boundary was present in 10% of eyes. Atrophic disciform scars developed in 6% of eyes

*CNV*, choroidal neovascularization; *GA*, geographic atrophy; *IQR*, interquartile range; *MNV*, macular neovascularization; *NA*, not applicable; *nAMD*, neovascular age-related macular degeneration; *OCT*, optical coherence tomography; *PRN*, *pro re nata*; *RAP*, retinal angiomatous proliferation; *RPE*, retinal pigment epithelial; *SD*, standard deviation; *SE*, standard error; *T&E*, treat-and-extend; *VA*, visual acuity; *VEGF*, vascular endothelial growth factor

**Table 3:** Imaging studies and reports

Citation	Study design/publication type	Indication	Types of imaging investigated	*n*	Key findings
Corbelli et al. (2017) [29]	Prospective, observational, single-center study	GA	FAF, en face structural OCT, OCT-A	47 eyes	OCT-A showed the greatest intraclass correlation coefficient for the detection of GA when compared to FAF. Interobserver agreement was higher in OCT-A compared to FAF for the detection of foveal involvement
Holz et al. (2017) [25]	Consensus meeting reports	CNV/GA	NA	NA	Color fundus photo, confocal FAF (blue light excitation), near-infrared reflectance, and SD- or SS-OCT volume scans should be performed for research in GA. FA may be required to detect incident neovascularization at baseline and at the final outcome point. For research in nAMD, SD- or SS-OCT volume scans should be performed at every visit. FA, FAF, color fundus photo, and NIR are recommended at baseline and at selected follow-up visits. The use of ICGA may be reasonable in eyes with RAP or PCV
Quinn et al. (2018) [28]	Retrospective, observational study	Hard drusen, soft drusen, reticular pseudodrusen, GA, choroidal neovascularization, naevus, epiretinal membrane, hemorrhage	CFP, IR, OCT	640 eyes	When using infrared reflectance combined with OCT, sensitivity and specificity for GA detection were 95.5% and 99.0%, respectively. For CNV detection, sensitivity and specificity were 80.0% and 99.8%, respectively
Nesper et al. (2018) [31]	Retrospective, observational, single-center study	GA	En face OCT, OCT-A	40 eyes	Medium-sized choroidal vessels were displaced anteriorly in areas of GA and visualized in the outer retinal slab of OCT-A. Cross-sectional OCT-A is needed to confirm the location of flow to avoid misdiagnosis of CNV
Massamba et al. (2019) [26]	Retrospective, observational, single-center study	nAMD with/without GA	FA, FAF, SDOCT, ICGA	97 eyes	SD-OCT image analysis demonstrated greater sensitivity than FAF in the identification of GA in patients treated for exudative AMD
Corvi et al. (2021) [30]	Prospective, observational, single-center study	Simultaneous CNV and GA/GA alone	FA, ICGA, OCT, OCT-A	42 patients	Manual segmentation on OCT-A allowed detection of MNV in 95.2% of eyes with macular atrophy and MNV and in 4.7% of eyes with GA, showing high specificity (95.2%) and sensitivity (95.2%)

*AMD*, age-related macular degeneration; *CFP*, color fundus photography; *CNV*, choroidal neovascularization; *FA*, fluorescein angiography; *FAF*, fundus autofluorescence; *GA*, geographic atrophy; *ICGA*, indocyanine green angiography; *IR*, infrared reflectance; *MNV*, macular neovascularization; *NA*, not applicable; *nAMD*, neovascular age-related macular degeneration; *NIR*, near-infrared reflectance; *OCT*, optical coherence tomography; *OCT-A*, OCT angiography; *PCV*, polypoidal choroidal vasculopathy; *RAP*, retinal angiomatous proliferation; *SD-OCT*, spectral-domain OCT; *SS-OCT*, swept-source OCT

**Table 4:** Risk factor of GA or CNV

Citation	Study design	Eyes studied	*n*	Follow-up	Key findings
Alexandre de Amorim Garcia Filho et al. (2013) [27]	Retrospective, observational, single-center study	Eyes with drusenoid PED	16 eyes	Mean 23.8 months (SD, 14.8 months)	Drusenoid PED progressed GA in 5 eyes (31.25%) and CNV in 2 eyes (12.5%)
Abdelfattah et al. (2016) [33]	Retrospective, observational, single-center study	Eyes with early or intermediate AMD	89 patients	24 months	4.5% of eyes developed CNV and 16.9% of eyes developed GA after 12 months. Larger drusen volume was a predictive factor for the development of GA or CNV
Finger et al. (2016) [35]	Prospective, observational study of a community-based cohort	Eyes with RPD	21,130 patients	NA	The prevalence of retinal pseudodrusen was 0.41%. Presence of GA increased odds of having RPD (OR, 153; 95% CI, 53–442) and CNV (OR, 90; 95% CI, 26–310)
Balaratnasingam et al. (2018) [34]	Retrospective, observational, multicenter study	Eyes with cuticular drusen	240 eyes	Mean 3.6 years (SD, 4.4 years)	Eyes with cuticular drusen progressed to CNV in 12.5% of patients and to GA in 25% of patients
Nassisi et al. (2019) [36]	Post hoc analysis of a multicenter study	Fellow eyes of the study eyes	501 eyes	24 months	Intraretinal hyperreflective foci, hyporeflective foci within drusenoid lesions, subretinal drusenoid deposits, and drusen volume of 0.03 mm^3^ or more were significantly associated with the development of late AMD including GA and MNV. The correlation remained significant when considering only the development of GA and MNV alone, except for drusen volume, which was not associated significantly with MNV development
Schmitz-Valckenberg et al. (2022) [38]	Retrospective, observational, single-center study	Eyes of patients with homozygous risk variants at both chromosome 1 and chromosome 10 or at either chromosome 1 or chromosome 10 alone	502 patients	NA	Eyes with early and intermediate AMD in patients with homozygous risk variants at both chromosome 1 and chromosome 10 and those with risk variants at chromosome 10 alone were 3.3 and 2.6 times more likely to develop late-stage AMD than patients with risk variants at chromosome 1 alone. This difference was mostly associated with conversion to macular neovascularization

*AMD*, age-related macular degeneration; *CI*, confidence interval; *CNV*, choroidal neovascularization; *GA*, geographic atrophy; *MNV*, macular neovascularization; *NA*, not applicable; *OR*, odds ratio; *PED*, pigment epithelial detachment; *RPD*, reticular pseudodrusen; *SD*, standard deviation

**Table 5:** Protection against GA development

Citation	Study design	Indication	*n*	Treatment	Follow-up	Key findings
Christenbury et al. (2018) [16]	Retrospective, observational, multicenter study	Type 1 MNV	27 patients	Aflibercept/ranibizumab/bevacizumab	Mean 63 months (SD, 24.5)	59.3% of eyes developed macular atrophy predominantly eccentric to PED, whereas 11.1% of eyes developed macular atrophy predominantly overlying PED. Expansion of macular atrophy was predominantly eccentric to the PED compared with atrophy overlying the PED
Pfau et al. (2020) [45]	Prospective, observational, single-center study	Late AMD	98 eyes	NA	1.17 years (IQR, 1.01–1.55)	Progression of macular atrophy was reduced in the presence of, compared with in the absence of, quiescent and exudative type 1 CNV

*AMD*, age-related macular degeneration; *CNV*, choroidal neovascularization; *GA*, geographic atrophy; *IQR*, interquartile range; *MNV*, macular neovascularization; *NA*, not applicable; *PED*, pigment epithelial detachment; *SD*, standard deviation

Recommendations for the diagnosis and assessment of simultaneous GA and CNV/MNV were developed by the authors and subsequently reviewed, commented upon, and endorsed by a majority of the Vision Academy membership, as previously described [7]. For each proposed recommendation, respondents were asked to rate their agreement using a 5-point categorical scale: ‘strongly agree’, ‘agree’, ‘neither agree nor disagree’, ‘disagree’, and ‘strongly disagree’. Responses from more than 50% of the Academy were required for the survey to be valid. To assess any influence of the healthcare system on the survey responses, respondents were additionally asked for the reimbursement status of treatment in their country of practice (reimbursed, ‘out-of-pocket’, or a combination of the two). Biases were assessed using χ^2^. Endorsement was established if 50% or more of respondents indicated that they agreed or strongly agreed with a recommendation; consensus was considered “strong” if greater than 75% of respondents agreed or strongly agreed. The list of Vision Academy members who contributed to the recommendations is provided in the Acknowledgments.

## Discussion/Observations

### Epidemiology

#### Incidence of simultaneous GA and CNV/MNV

Studies have reported the incidence of CNV/MNV subsequent to GA. The Macular Photocoagulation Study Group observed that CNV/MNV progressed in eyes with simultaneous GA, although the rate of CNV development did not differ significantly between eyes with and eyes without GA [8]. In a large retrospective cohort analysis of a multicenter electronic medical record database, Chakravarthy et al. reported a CNV/MNV incidence rate of 7.4% per patient-year in eyes with GA during a mean follow-up period of 1.4 years [4]. In a post hoc analysis of the AREDS2 study, CNV/MNV was observed in 13.8% of eyes with GA over a mean follow-up period of 4.1 years [5].

The incidence of macular atrophy following CNV/MNV has been reported during the long-term follow-up of patients receiving anti–vascular endothelial growth factor (VEGF) treatment for CNV/MNV. In the SEVEN-UP study, a long-term follow-up study of patients from the MARINA and ANCHOR studies, 98% of eyes showed macular atrophy during the mean follow-up period of 7.3 years, which was correlated with poor visual outcomes [9]. However, the rates of macular atrophy at baseline and the incidence rate of newly formed macular atrophy were not disclosed. The incidence rate of newly formed macular atrophy at 24 months in eyes with CNV/MNV treated with anti-VEGF therapies was 29.4% in the HARBOR study [10, 11], 24.4% in the IVAN study [12], and 37% in the RIVAL study [13]. A retrospective observational study reported that the occurrence of macular atrophy in 292 eyes increased from 16% at baseline to 42.5% after 5 years [14]. In patients with simultaneous GA and CNV/MNV at baseline, enlargement of GA has been reported during anti-VEGF therapy or follow-up for CNV/MNV [12, 14–19]. A post hoc analysis of the IVAN study reported that 64.3% of eyes with simultaneous GA and CNV/MNV at baseline showed GA expansion of 20% or more during 24 months of follow-up [12]. A post hoc analysis of the CATT study reported that eyes with retinal angiomatous proliferation (RAP) or type 3 MNV demonstrated GA (31.8%) more than eyes without RAP (19.4%) [20].

#### Quiescent CNV/MNV in GA

A recent study using optical coherence tomography angiography (OCT-A) reported the detection of subclinical CNV/MNV in 11.0–16.0% of eyes with GA [6, 21]. Thus, multimodal imaging, including en face and cross-sectional optical coherence tomography (OCT) and OCT-A, could reveal higher incidence or prevalence rates of simultaneous GA and CNV/MNV than have previously been reported.

#### Population differences

When discussing simultaneous GA and CNV/MNV, it is important to note that the incidence and prevalence of GA vary between racial groups and geographical regions. A systematic review and meta-analysis by Wong et al. reported a higher prevalence of GA in European (1.11%) compared to African (0.14%), Asian (0.21%), and Hispanic (0.16%) patients [22], while Rim et al. reported that the prevalence of GA was higher in South Asia (0.382%) compared with East Asia (0.076%) [23]. In contrast, an Asian cohort study from Singapore reported that macular atrophy increased from 12.5 to 18.8% in non-polypoidal choroidal vasculopathy (PCV) eyes and 6.7–15.6% in PCV eyes during the 1-year follow-up period under anti-VEGF therapy [24]. Thus, population differences should be taken into account when discussing simultaneous GA and CNV/MNV and when planning research.

### Assessment and diagnosis

Multimodal imaging is recommended for the detection of both macular atrophy and CNV/MNV; however, CNV/MNV or a fibrotic scar may mask macular atrophy, especially at initial presentation, making the diagnosis of GA difficult. The consensus Classification of Atrophy Meeting [25] proposed that color fundus photo, confocal fundus autofluorescence (FAF) using blue light excitation, near-infrared reflectance (NIR), and spectral-domain OCT (SD-OCT) or swept-source OCT (SS-OCT) volume scans should be performed for research studies in GA. For studies in neovascular age-related macular degeneration (nAMD), SD- or SS-OCT volume scans, at least covering the macular area and with a maximum distance between scans no greater than 120 μm, should be performed at every visit. Fluorescein angiography, FAF, color fundus photo, and NIR are recommended at baseline and at selected follow-up visits. The need for an indocyanine green angiography examination depends on the type of lesion but may be advisable in eyes suspected to have or having retinal-choroidal anastomosis or PCV [25].

Where GA is present, the loss of RPE and lipofuscin blue light excitation FAF signal is indicating a loss of retinal sensitivity. The quality of the FAF signal can also be influenced by optic media opacities or obstruction by macular pigment, hemorrhages, or hard exudates [25]. Cross-sectional or en face SD-OCT image analysis has been reported to offer greater sensitivity than FAF in the identification of macular atrophy in patients treated for exudative AMD because of the increased penetration of light into the choroid [26, 27]. NIR provides complementary information when combined with FAF or OCT [25, 28]. OCT-A is also useful for the detection of both macular atrophy and MNV/CNV. In a study by Corbelli et al., macular atrophy was detected as areas with a lack of choriocapillaris with improved visualization of the choroid. Interobserver agreement on the detection of foveal involvement was greater with OCT-A than with FAF [29]. In another study, manual segmentation on OCT-A allowed the detection of the MNV in 95.2% of eyes with GA and simultaneous CNV/MNV, and in 4.7% of eyes with GA, with high levels of specificity (95.2%) and sensitivity (95.2%) [30]. It has been noted that medium-sized choroidal vessels are displaced anteriorly in areas of macular atrophy and visualized in the outer retinal slab of OCT-A. Thus, cross-sectional OCT-A is needed to confirm the location of flow to avoid misdiagnosis of CNV [31].

### Clinical characteristics of simultaneous GA and CNV/MNV

A study by Saade et al. found that patients with simultaneous GA and CNV/MNV were older and had higher prevalence of late AMD in the fellow eyes than patients with GA alone or CNV alone [32]. Several clinical characteristics have been identified as risk factors for both GA and CNV/MNV. Increased drusen volume, drusenoid pigment epithelial detachment (PED), cuticular drusen, and subretinal drusenoid deposits are known to pre-exist or coexist with both GA and CNV/MNV [27, 33–35]. A post hoc analysis of the HARBOR study reported that intraretinal hyperreflective foci, hyporeflective foci within drusenoid lesions, subretinal drusenoid deposits, and drusen volume of 0.03 mm^3^ or more were significantly associated with the development of late AMD, including GA and CNV/MNV [36].

### Genetic risk factors for simultaneous GA and CNV/MNV

Grob et al. compared clinical presentation and genotypes in patients with combined GA/CNV vs. patients with either CNV alone or GA alone. They reported no statistical difference in allelic frequency of high-temperature requirement factor A1 (*HTRA1*), complement factor H (*CFH*), complement factor 3 (*C3*), and complement factor 2, suggesting that CNV and GA may be a continuum of AMD [37]. The study by Saade et al. also reported no significant difference in *CFH* and age-related maculopathy susceptibility 2 (*ARMS2*) risk alleles between patients with combined GA/CNV and those with GA or CNV alone [32], further supporting the suggestion that GA and CNV/MNV may not be separate diseases. A study by Schmitz-Valckenberg et al. investigated the course of AMD in patients with genetic risk allele *CFH*–complement factor H-related 5 at chromosome 1 and *ARMS2/HTRA1* at chromosome 10. They found that eyes with early and intermediate AMD in patients who were homozygous for risk variants at both chromosome 1 and chromosome 10 or at chromosome 10 alone were 3.3 and 2.6 times more likely to develop late-stage AMD, respectively, compared with participants with risk variants at chromosome 1 alone. This difference was mostly associated with progression to MNV [38].

The reported findings that GA and CNV/MNV share common clinical and genetic risk factors further supports the suggestion that GA and CNV/MNV could be a continuum of late AMD.

### Treatment and prognosis

#### Development of macular/GA during anti-VEGF treatment

No clinical trial to date has focused on the treatment of simultaneous GA and CNV/MNV. Currently, patients with CNV/MNV with or without macular atrophy are treated with anti-VEGF therapy [39]. However, macular atrophy that is either present at baseline or develops during treatment is associated with poor visual outcomes following anti-VEGF therapy [9, 40, 41]. A number of studies have investigated whether anti-VEGF therapy for CNV/MNV could accelerate new development or enlargement of macular atrophy, with several publications reporting no association between macular atrophy enlargement and anti-VEGF treatment [5, 19, 42]. A post hoc analysis of the IVAN study showed that with respect to incident intralesional macular atrophy, eyes treated with bevacizumab did not differ from those treated with ranibizumab, and the number of treatment cycles had no effect [12]. In a post hoc analysis of the HARBOR study, neither the rate of enlargement of the macular atrophy area nor time to detection of macular atrophy differed with the ranibizumab dose (0.5 mg vs. 2.0 mg) or frequency of treatment (monthly vs. *pro re nata*) [11]. In another HARBOR study post hoc analysis, monthly vs. *pro re nata* treatment trended towards an association with macular atrophy (hazard ratio, 1.29; 95% confidence interval, 0.99–1.68), but statistical significance was not reached [10]. No significant differences in the rate of development or growth of macular atrophy over 24 months were observed between ranibizumab and aflibercept in patients with nAMD treated using a treat-and-extend regimen [13]. In a study by Arevalo et al., the presence of macular atrophy in eyes with CNV/MNV did not affect the number of anti-VEGF injections required [14].

A post hoc analysis of the ANCHOR and MARINA studies, in which the study eye received high-frequency ranibizumab while the fellow eye was untreated, reported that delaying anti-VEGF therapy resulted in poor vision outcomes and progression of macular atrophy [18]. On the other hand, the Observing Fibrosis, Macular Atrophy and Subretinal Highly Reflective Material, Before and After Intervention with anti-VEGF Treatment (FASBAT) study compared the increase of macular atrophy prevalence before and after initiation of anti-VEGF therapy and reported that early diagnosis of nAMD does not influence the prevalence of macular atrophy [43]. Further study is needed to confirm the effect of inhibition of the VEGF pathway on RPE damage.

Several risk factors for the development of macular atrophy during anti-VEGF therapy have been identified. These include the development of fibrotic scars and the presence of type 3 neovascularization [17, 20]. Post hoc analyses of the HARBOR study also identified at baseline the presence of intraretinal cysts, GA in fellow eyes, older age, higher central drusen volume, lower choroidal thickness, nascent atrophy (loss of outer retinal layers with no loss or thinning of RPE), subretinal drusenoid deposits, increased central foveal thickness, and atrophy in the fellow eye as risk factors for the development of macular atrophy [10, 44].

A number of protective factors for macular atrophy development during anti-VEGF therapy have also been reported. A post hoc analysis of the HARBOR study reported that subretinal fluid at baseline was associated with a decreased risk of macular atrophy development [10]. This was also the case in a post hoc analysis of the IVAN study, which reported that eyes with predominantly classic CNV at baseline, subretinal fluid at the final visit, or PED at the final visit had a reduced risk of incident intralesional macular atrophy by the final visit compared with eyes without these features [12]. The AREDS2 Report 24 reported that, in eyes with recent appearance of macular atrophy, the subsequent appearance of exudative nAMD was associated with a reduced rate of enlargement of the macular atrophy [5]. In another study, the colocalization of macular atrophy with quiescent and exudative type 1 CNV was found to hinder macular atrophy progression compared with macular atrophy without colocalizing type 1 CNV [45]. In an assessment of macular atrophy lesions and the location of type 1 CNV/MNV, it was found that 59.3% of eyes developed macular atrophy predominantly eccentric to the type 1 CNV/MNV lesions, whereas 11.1% of eyes developed macular atrophy predominantly overlying the type 1 CNV/MNV lesion [16]. Thus, type 1 CNV/MNV may play a protective role in slowing the progression of macular atrophy. Understanding the mechanisms through which a healthy RPE is maintained in type 1 CNV may be key to identifying a new treatment strategy for simultaneous GA and CNV/MNV.

#### Development of new CNV/MNV during clinical trials for GA

The complement pathway is reported to be involved in the development of GA. A phase 1 trial of NGM621, an immunoglobulin G1 monoclonal antibody with 2-point mutations in the Fc region, in the inhibition of C3 in patients with GA secondary to AMD found this to be a safe and tolerable drug with no obvious impact on newly formed CNV/MNV [46]. However, the CATALINA phase 2 trial of NGM621 for eyes with GA recently announced that it had not met its primary endpoint of prevention of the enlargement of the GA lesion [47]. Pegcetacoplan is another inhibitor of C3 cleavage that has recently been approved by the US Food and Drug Administration. The FILLY trial, a phase 2 study of pegcetacoplan in the treatment of GA, showed a reduction of GA growth [48]. However, a post hoc analysis of the FILLY trial reported a dose-dependent increased incidence of CNV/MNV in the treatment group compared with the sham injection group [48, 49]. In the FILLY trial, pegcetacoplan was administered monthly or bimonthly for 12 months, followed by a 6-month off-treatment period. The safety report revealed exudative changes by the end of the 18 months in 18 out of 86 patients (20.9%) treated with monthly pegcetacoplan, 7 out of 79 patients (8.9%) treated with bimonthly pegcetacoplan, and 1 out of 81 patients (1.2%) receiving sham injections. The mean time to exudative changes was 8.5 months [49]. Exudative AMD was identified at a more frequent rate in the pegcetacoplan arms than with the sham arm and appears to be dose dependent. Furthermore, the safety assessment reported that exudative AMD in the fellow eye and double-layer sign in the study eye tended to increase the risk of exudative changes associated with pegcetacoplan treatment [49]. The OAKS and DERBY phase 3 clinical trials of pegcetacoplan in patients with GA reported that monthly or every-other-month intravitreal injections of pegcetacoplan significantly slow the progression of GA compared to sham arms [50]. However, dose-dependent exudative AMD was reported at 24 months in 11% (the OAKS trial) and 13% (the DERBY trial) of eyes treated with monthly pegcetacoplan, 8% (the OAKS trial) and 6% (the DERBY trial) of eyes treated with bimonthly pegcetacoplan, and 2% (the OAKS trial) and 4% (the DERBY trial) of eyes treated with sham injections [51]. Avacincaptad pegol (ACP) is an inhibitor of complement C5 cleavage for the treatment of GA. The GATHER1 trial, a phase 2/3 trial for the treatment of GA, reported the significant reduction in GA growth at 18 months in patients treated with ACP 2 mg or 4 mg compared to those receiving sham injections. However, the development of dose-dependent MNV was observed in both groups treated with either ACP 2 mg (11.9%) or 4 mg (15.7%) compared to sham groups (2.7% and 2.4%) [52]. Furthermore, the GATHER2 trial, a phase 3 trial of ACP for the reduction of GA, reported reduced GA growth in patients treated with ACP 2 mg compared to patients treated with sham injections but found that 7% of patients treated with ACP 2 mg and 4% of patients treated with sham injections developed MNV [53]. Recently, the role of senescent cells has been implicated in the pathobiology of AMD. As of now, it is not clear how new therapeutics for GA interact with these cells and their function and if their inhibition could be a reason for increase in neovascularization [54].

### Vision Academy recommendations

The recommendations listed in this article were formulated by the authors and submitted to the entire Vision Academy membership for endorsement; 47 responses (including from the authors) were received. Overall, the recommendations were endorsed by 92.2% of the respondents (a response of ‘agree’ or ‘strongly agree’), with the level of endorsement for each individual recommendation ranging from 87.2 to 97.9%. The mean (range) rate of non-endorsement was 3.5% (0–6.4%) for a response of either ‘disagree’ or ‘strongly disagree’, and 4.3% (2.1–6.4%) for a response of ‘neither agree nor disagree’. (It should be noted that these recommendations represent the ideal scenario, and full implementation may not be possible in all clinics.) The recommendations are listed below and summarized in Table 6.

**Table 6:** Vision Academy recommendations

Clinical practice	Research
• Aware of potential coexistence and/or development of GA and CNV/MNV • Assessment for GA in patients being treated for CNV/MNV • Assessment for CNV/MNV in patients with GA • Multimodal imaging (FAF, NIR, cross-sectional OCT, and OCT-A) or at a minimum, cross-sectional OCT at each visit	• A detailed assessment for both GA and CNV/MNV (including quiescent CNV/MNV) • Multimodal imaging (FAF, NIR, cross-sectional OCT, and OCT-A) at each trial visit • Dye-based angiography at trial entry and at any timepoint when new CNV/MNV developing

*CNV*, choroidal neovascularization; *FAF*, fundus autofluorescence; *GA*, geographic atrophy; *MNV*, macular neovascularization; *NIR*, near-infrared reflectance; *OCT*, optical coherence tomography; *OCT-A*, OCT angiography

i) When managing AMD, both the clinician and patient should be aware of potential coexistence and/or development of GA and CNV/MNV. Clinicians should be vigilant and assess for GA in patients being treated for CNV/MNV and assess for CNV/MNV in patients with GA.
ii) *In clinical trials*, a detailed assessment for both GA and CNV/MNV (including quiescent CNV/MNV) with multimodal imaging consisting of FAF, NIR, cross-sectional OCT, and OCT-A is ideally recommended at each trial visit. In addition, dye-based angiography should be performed at trial entry and at any timepoint when there is development of new CNV/MNV.
iii) Multimodal imaging, including FAF, NIR, cross-sectional OCT, and OCT-A, would ideally be recommended at each visit *in clinical practice*. If this is not possible, at a minimum, cross-sectional OCT should be performed at each visit.

## Conclusions

The evidence that GA and CNV/MNV share common genetic risk factors and clinical characteristics, and that simultaneous GA and CNV/MNV is divided into two types – CNV/MNV subsequent to GA and macular atrophy subsequent to CNV/MNV – suggests that GA and CNV/MNV are part of a continuum of late-stage AMD. Thus, simultaneous GA and CNV/MNV should be considered in any discussion of the long-term management of late AMD and in research into new treatments for the disease. More data on the treatment of simultaneous GA and CNV/MNV, particularly real-world outcomes data collected in a systematic manner, are required.

## Electronic supplementary material

Below is the link to the electronic supplementary material.

Supplementary Material 1 (DOCX 353 KB)

## References

[1] Ferris FL III, Wilkinson CP, Bird A et al (2013) Clinical classification of age-related macular degeneration. Ophthalmology 120:844–851. 10.1016/j.ophtha.2012.10.036 23332590 10.1016/j.ophtha.2012.10.036 PMC11551519

[2] Spaide RF, Jaffe GJ, Sarraf D et al (2020) Consensus nomenclature for reporting neovascular age-related macular degeneration data: consensus on neovascular age-related macular degeneration nomenclature study group. Ophthalmology 127:616–636. 10.1016/j.ophtha.2019.11.004 31864668 10.1016/j.ophtha.2019.11.004 PMC11559632

[3] Sadda SR, Guymer R, Holz FG et al (2018) Consensus definition for atrophy associated with age-related macular degeneration on OCT: Classification of Atrophy Report 3. Ophthalmology 125:537–548. 10.1016/j.ophtha.2017.09.028 29103793 10.1016/j.ophtha.2017.09.028 PMC11366072

[4] Chakravarthy U, Bailey CC, Johnston RL et al (2018) Characterizing disease burden and progression of geographic atrophy secondary to age-related macular degeneration. Ophthalmology 125:842–849. 10.1016/j.ophtha.2017.11.036 29366564 10.1016/j.ophtha.2017.11.036

[5] Hwang CK, Agrón E, Domalpally A et al (2021) Progression of geographic atrophy with subsequent exudative neovascular disease in age-related macular degeneration: AREDS2 Report 24. Ophthalmol Retina 5:108–117. 10.1016/j.oret.2020.10.008 33075546 10.1016/j.oret.2020.10.008 PMC7870515

[6] Capuano V, Miere A, Querques L et al (2017) Treatment-naïve quiescent choroidal neovascularization in geographic atrophy secondary to nonexudative age-related macular degeneration. Am J Ophthalmol 182:45–55. 10.1016/j.ajo.2017.07.009 28734811 10.1016/j.ajo.2017.07.009

[7] Mathis T, Holz FG, Sivaprasad S et al (2023) Characterisation of macular neovascularisation subtypes in age-related macular degeneration to optimise treatment outcomes. Eye 37:1758–1765. 10.1038/s41433-022-02231-y 36104522 10.1038/s41433-022-02231-y PMC10275926

[8] Maguire MG, Bressler SB, Bresskr NM et al (1997) Risk factors for choroidal neovascularization in the second eye of patients with juxtafoveal or subfoveal choroidal neovascularization secondary to age-related macular degeneration. Arch Ophthalmol 115:741–747. 10.1001/archopht.1997.01100150743009 9194725 10.1001/archopht.1997.01100150743009

[9] Rofagha S, Bhisitkul RB, Boyer DS et al (2013) Seven-year outcomes in ranibizumab-treated patients in ANCHOR, MARINA, and HORIZON: a multicenter cohort study (SEVEN-UP). Ophthalmology 120:2292–2299. 10.1016/j.ophtha.2013.03.046 23642856 10.1016/j.ophtha.2013.03.046

[10] Sadda SR, Tuomi LL, Ding B et al (2018) Macular atrophy in the HARBOR study for neovascular age-related macular degeneration. Ophthalmology 125:878–886. 10.1016/j.ophtha.2017.12.026 29477692 10.1016/j.ophtha.2017.12.026

[11] Gune S, Abdelfattah NS, Karamat A et al (2020) Spectral-domain OCT–based prevalence and progression of macular atrophy in the HARBOR study for neovascular age-related macular degeneration. Ophthalmology 127:523–532. 10.1016/j.ophtha.2019.09.030 31718842 10.1016/j.ophtha.2019.09.030

[12] Bailey C, Scott LJ, Rogers CA et al (2019) Intralesional macular atrophy in anti–vascular endothelial growth factor therapy for age-related macular degeneration in the IVAN trial. Ophthalmology 126:75–86. 10.1016/j.ophtha.2018.07.013 30301555 10.1016/j.ophtha.2018.07.013

[13] Gillies MC, Hunyor AP, Arnold JJ et al (2020) Macular atrophy in neovascular age-related macular degeneration: a randomized clinical trial comparing ranibizumab and aflibercept (RIVAL study). Ophthalmology 127:198–210. 10.1016/j.ophtha.2019.08.023 31619357 10.1016/j.ophtha.2019.08.023

[14] Arevalo JF, Lasave AF, Wu L et al (2016) Intravitreal bevacizumab for choroidal neovascularization in age-related macular degeneration: 5-year results of the Pan-American Collaborative Retina Study Group. Retina 36:859–867. 10.1097/IAE.0000000000000827 26529555 10.1097/IAE.0000000000000827

[15] Tanaka E, Chaikitmongkol V, Bressler SB, Bressler NM (2015) Vision-threatening lesions developing with longer-term follow-up after treatment of neovascular age-related macular degeneration. Ophthalmology 122:153–161. 10.1016/j.ophtha.2014.07.046 25283060 10.1016/j.ophtha.2014.07.046

[16] Christenbury JG, Phasukkijwatana N, Gilani F et al (2018) Progression of macular atrophy in eyes with type 1 neovascularization and age-related macular degeneration receiving long-term intravitreal anti-vascular endothelial growth factor therapy: an optical coherence tomographic angiography analysis. Retina 38:1276–1288. 10.1097/IAE.0000000000001766 28723848 10.1097/IAE.0000000000001766

[17] Daniel E, Pan W, Ying GS et al (2018) Development and course of scars in the Comparison of Age-Related Macular Degeneration Treatments Trials. Ophthalmology 125:1037–1046. 10.1016/j.ophtha.2018.01.004 29454660 10.1016/j.ophtha.2018.01.004 PMC6015772

[18] Bhisitkul RB, Desai S, Boyer DS et al (2016) Fellow eye comparisons for 7-year outcomes in ranibizumab-treated AMD subjects from ANCHOR, MARINA, and HORIZON (SEVEN-UP Study). Ophthalmology 123:1269–1277. 10.1016/j.ophtha.2016.01.033 26996339 10.1016/j.ophtha.2016.01.033

[19] Siedlecki J, Koch C, Schworm B et al (2021) Enlargement rate of geographic atrophy before and after secondary CNV conversion with associated anti-VEGF treatment. BMC Ophthalmol 21:1–8. 10.1186/s12886-020-01766-6 33402147 10.1186/s12886-020-01766-6 PMC7784328

[20] Daniel E, Shaffer J, Ying GS et al (2016) Outcomes in eyes with retinal angiomatous proliferation in the Comparison of Age-Related Macular Degeneration Treatments Trials (CATT). Ophthalmology 123:609–616. 10.1016/j.ophtha.2015.10.034 26681392 10.1016/j.ophtha.2015.10.034 PMC4766028

[21] de Oliveira Dias JR, Zhang Q, Garcia JMB et al (2018) Natural history of subclinical neovascularization in nonexudative age-related macular degeneration using swept-source OCT angiography. Ophthalmology 125:255–266. 10.1016/j.ophtha.2017.08.030 28964581 10.1016/j.ophtha.2017.08.030 PMC11402511

[22] Wong WL, Su X, Li X et al (2014) Global prevalence of age-related macular degeneration and disease burden projection for 2020 and 2040: a systematic review and meta-analysis. Lancet Glob Health 2:e106–e116. 10.1016/s2214-109x(13)70145-1 25104651 10.1016/S2214-109X(13)70145-1

[23] Rim TH, Kawasaki R, Tham YC et al (2020) Prevalence and pattern of geographic atrophy in Asia: the Asian Eye Epidemiology Consortium. Ophthalmology 127:1371–1381. 10.1016/j.ophtha.2020.04.019 32344073 10.1016/j.ophtha.2020.04.019

[24] Cheung CMG, Grewal DS, Teo KYC et al (2019) The evolution of fibrosis and atrophy and their relationship with visual outcomes in Asian persons with neovascular age-related macular degeneration. Ophthalmol Retina 3:1045–1055. 10.1016/j.oret.2019.06.002 31444144 10.1016/j.oret.2019.06.002

[25] Holz FG, Sadda SR, Staurenghi G et al (2017) Imaging protocols in clinical studies in advanced age-related macular degeneration: recommendations from Classification of Atrophy Consensus Meetings. Ophthalmology 124:464–478. 10.1016/j.ophtha.2016.12.002 28109563 10.1016/j.ophtha.2016.12.002

[26] Massamba N, Sellam A, Butel N et al (2019) Use of fundus autofluorescence combined with optical coherence tomography for diagnose of geographic atrophy in age-related macular degeneration. Med Hypothesis Discov Innov Ophthalmol 8:298–305 31788492 PMC6778681

[27] Alexandre de Amorim Garcia Filho C, Yehoshua Z, Gregori G et al (2013) Spectral-domain optical coherence tomography imaging of drusenoid pigment epithelial detachments. Retina 33:1558–1566. 10.1097/IAE.0b013e318285cbd2 23584692 10.1097/IAE.0b013e318285cbd2

[28] Quinn NB, Chakravarthy U, Muldrew KA et al (2018) Confocal infrared imaging with optical coherence tomography provides superior detection of a number of common macular lesions compared to colour fundus photography. Ophthalmic Physiol Opt 38:574–583. 10.1111/opo.12592 30575074 10.1111/opo.12592

[29] Corbelli E, Sacconi R, Rabiolo A et al (2017) Optical coherence tomography angiography in the evaluation of geographic atrophy area extension. Invest Ophthalmol Vis Sci 58:5201–5208. 10.1167/iovs.17-22508 29049720 10.1167/iovs.17-22508

[30] Corvi F, Cozzi M, Invernizzi A et al (2021) Optical coherence tomography angiography for detection of macular neovascularization associated with atrophy in age-related macular degeneration. Graefes Arch Clin Exp Ophthalmol 259:291–299. 10.1007/s00417-020-04821-6 32620993 10.1007/s00417-020-04821-6

[31] Nesper PL, Lutty GA, Fawzi AA (2018) Residual choroidal vessels in atrophy can masquerade as choroidal neovascularization on optical coherence tomography angiography: introducing a clinical and software approach. Retina 38:1289–1300. 10.1097/IAE.0000000000001863 29059100 10.1097/IAE.0000000000001863 PMC5910298

[32] Saade C, Ganti B, Marmor M et al (2014) Risk characteristics of the combined geographic atrophy and choroidal neovascularisation phenotype in age-related macular degeneration. Br J Ophthalmol 98:1729–1732. 10.1136/bjophthalmol-2014-305005 25091949 10.1136/bjophthalmol-2014-305005 PMC4233165

[33] Abdelfattah NS, Zhang H, Boyer DS et al (2016) Drusen volume as a predictor of disease progression in patients with late age-related macular degeneration in the fellow eye. Invest Ophthalmol Vis Sci 57:1839–1846. 10.1167/iovs.15-18572 27082298 10.1167/iovs.15-18572

[34] Balaratnasingam C, Cherepanoff S, Dolz-Marco R et al (2018) Cuticular drusen: clinical phenotypes and natural history defined using multimodal imaging. Ophthalmology 125:100–118. 10.1016/j.ophtha.2017.08.033 28964580 10.1016/j.ophtha.2017.08.033

[35] Finger RP, Chong E, McGuinness MB et al (2016) Reticular pseudodrusen and their association with age-related macular degeneration: The Melbourne Collaborative Cohort Study. Ophthalmology 123:599–608. 10.1016/j.ophtha.2015.10.029 26681391 10.1016/j.ophtha.2015.10.029

[36] Nassisi M, Lei J, Abdelfattah NS et al (2019) OCT risk factors for development of late age-related macular degeneration in the fellow eyes of patients enrolled in the HARBOR study. Ophthalmology 126:1667–1674. 10.1016/j.ophtha.2019.05.016 31281056 10.1016/j.ophtha.2019.05.016

[37] Grob S, Luo J, Hughes G et al (2012) Genetic analysis of simultaneous geographic atrophy and choroidal neovascularization. Eye (Lond) 26:1106–1113. 10.1038/eye.2012.107 22699975 10.1038/eye.2012.107 PMC3420042

[38] Schmitz-Valckenberg S, Fleckenstein M, Zouache MA et al (2022) Progression of age-related macular degeneration among individuals homozygous for risk alleles on chromosome 1 (*CFH-CFHR5*) or chromosome 10 (*ARMS2/HTRA1*) or both. JAMA Ophthalmol 140:252–260. 10.1001/jamaophthalmol.2021.6072 35113155 10.1001/jamaophthalmol.2021.6072 PMC8814975

[39] Amaro MH, Roller AB (2012) Intravitreal ranibizumab and bevacizumab therapy for choroidal neovascularization in age-related macular degeneration with extensive pre-existing geographic atrophy. Arq Bras Oftalmol 75:273–276. 10.1590/s0004-27492012000400011 23258660 10.1590/s0004-27492012000400011

[40] Cohen SY, Oubraham H, Uzzan J et al (2012) Causes of unsuccessful ranibizumab treatment in exudative age-related macular degeneration in clinical settings. Retina 32:1480–1485. 10.1097/IAE.0b013e318240a516 22258164 10.1097/IAE.0b013e318240a516

[41] Ying GS, Maguire MG, Daniel E et al (2015) Association of baseline characteristics and early vision response with 2-year vision outcomes in the Comparison of AMD Treatments Trials (CATT). Ophthalmology 122:2523–2531e2521. 10.1016/j.ophtha.2015.08.015 26383996 10.1016/j.ophtha.2015.08.015 PMC4658285

[42] Casalino G, Arrigo A, Introini U et al (2021) Clinical course of treated choroidal neovascularization in eyes with pre-existing geographic atrophy: case series and reappraisal of the literature. Curr Eye Res 46:988–994. 10.1080/02713683.2020.1849730 33174459 10.1080/02713683.2020.1849730

[43] Gale RP, Airody A, Sivaprasad S et al (2024) Improved structure and function in early-detected second-eye neovascular age-related macular degeneration: FASBAT/early detection of neovascular age-related macular degeneration report 1. Ophthalmol Retina 8:545–552. 10.1016/j.oret.2023.12.012 38171416 10.1016/j.oret.2023.12.012

[44] Sadda SR, Abdelfattah NS, Lei J et al (2020) Spectral-domain OCT analysis of risk factors for macular atrophy development in the HARBOR study for neovascular age-related macular degeneration. Ophthalmology 127:1360–1370. 10.1016/j.ophtha.2020.03.031 32402555 10.1016/j.ophtha.2020.03.031

[45] Pfau M, Möller PT, Künzel SH et al (2020) Type 1 choroidal neovascularization is associated with reduced localized progression of atrophy in age-related macular degeneration. Ophthalmol Retina 4:238–248. 10.1016/j.oret.2019.09.016 31753808 10.1016/j.oret.2019.09.016

[46] Wykoff CC, Hershberger V, Eichenbaum D et al (2022) Inhibition of complement factor 3 in geographic atrophy with NGM621: phase 1 dose-escalation study results. Am J Ophthalmol 235:131–142. 10.1016/j.ajo.2021.08.018 34509438 10.1016/j.ajo.2021.08.018

[47] NGM Bio (2022) NGM Bio announces topline results from the CATALINA Phase 2 trial of NGM621 in patients with geographic atrophy (GA) secondary to age-related macular degeneration. https://ir.ngmbio.com/node/9586/pdf. Accessed 9 Apr 2025

[48] Liao DS, Grossi FV, El Mehdi D et al (2020) Complement C3 inhibitor pegcetacoplan for geographic atrophy secondary to age-related macular degeneration: a randomized phase 2 trial. Ophthalmology 127:186–195. 10.1016/j.ophtha.2019.07.011 31474439 10.1016/j.ophtha.2019.07.011

[49] Wykoff CC, Rosenfeld PJ, Waheed NK et al (2021) Characterizing new-onset exudation in the randomized phase 2 FILLY trial of complement inhibitor pegcetacoplan for geographic atrophy. Ophthalmology 128:1325–1336. 10.1016/j.ophtha.2021.02.025 33711380 10.1016/j.ophtha.2021.02.025

[50] Chiang A, Bliss C, Ribeiro R (2023) Assessment of geographic atrophy (GA) lesion progression in the phase 3 OAKS and DERBY trials. Invest Ophthalmol Vis Sci 64:986

[51] Heier JS, Lad EM, Holz FG et al (2023) Pegcetacoplan for the treatment of geographic atrophy secondary to age-related macular degeneration (OAKS and DERBY): two multicentre, randomised, double-masked, sham-controlled, phase 3 trials. Lancet 402:1434–1448. 10.1016/S0140-6736(23)01520-9 37865470 10.1016/S0140-6736(23)01520-9

[52] Patel SS, Lally DR, Hsu J et al (2023) Avacincaptad pegol for geographic atrophy secondary to age-related macular degeneration: 18-month findings from the GATHER1 trial. Eye 37:3551–3557. 10.1038/s41433-023-02497-w 36964259 10.1038/s41433-023-02497-w PMC10686386

[53] Khanani AM, Patel SS, Staurenghi G et al (2023) Efficacy and safety of avacincaptad pegol in patients with geographic atrophy (GATHER2): 12-month results from a randomised, double-masked, phase 3 trial. Lancet 402:1449–1458. 10.1016/S0140-6736(23)01583-0 37696275 10.1016/S0140-6736(23)01583-0

[54] Malek G, Campisi J, Kitazawa K et al (2022) Does senescence play a role in age-related macular degeneration? Exp Eye Res 225:109254. 10.1016/j.exer.2022.109254 36150544 10.1016/j.exer.2022.109254 PMC10032649

