# Supplementary material for: Simultaneous GA and CNV/MNV: incidence, characteristics, and treatments
Source: Graefes Arch Clin Exp Ophthalmol. 2025 Apr 14;263(5):1197–212. doi: 10.1007/s00417-024-06721-5 (PMC12149262; doi:10.1007/s00417-024-06721-5)
Supplement: Supplementary file 1 — Supplementary Material 1 (DOCX 353 KB) [file 417_2024_6721_MOESM1_ESM.docx]

**Simultaneous GA and CNV/MNV: incidence, characteristics, and treatments**

**Subtitle: A review**

Keiko Kataoka, MD, PhD^1^; Richard Gale, FRCP, FRCOphth, MEd, PhD^2^; Xiaoxin Li, MD^3^; Figen Şermet, MD^4^; Cynthia X. Qian, MDCM, FRCSC, DABO^5^; Chui Ming Gemmy Cheung, MBBS, FRCOphth, FAMS, MCI^6,7^; Miltiadis K. Tsilimbaris, MD, PhD^8^; Igor Kozak, MD, PhD^9,10^

*^1^Kyorin University School of Medicine, Tokyo, Japan; ^2^Hull York Medical School, University of York and York Teaching Hospital NHS Foundation Trust, York, UK; ^3^Eye Center and Eye Institute of Peking University People’s Hospital, Beijing, China; ^4^Ophthalmology Department, Ankara University School of Medicine, Ankara, Turkey; ^5^Department of Ophthalmology, University of Montreal, Montreal, Canada; ^6^Singapore Eye Research Institute, Singapore National Eye Centre, Singapore; ^7^Duke-NUS Medical School, National University of Singapore, Singapore;* *^8^University of Crete Medical School, Crete, Greece; ^9^Moorfields Eye Hospital Centre, Abu Dhabi, United Arab Emirates; ^10^University of Arizona-Tucson, Tucson, Arizona, USA.*

**Correspondence:** Igor Kozak, MD, PhD, Moorfields Eye Hospital Centre, Abu Dhabi, United Arab Emirates. E-mail: ikozak@arizona.edu. Tel: +97126356161

**Supplementary Methods**

The detailed search strategy is shown in Supplementary Table 1. A single reviewer (Keiko Kataoka) screened titles and abstracts for eligibility. Articles not discussing geographic atrophy or choroidal neovascularization/macular neovascularization related to age-related macular degeneration were excluded. The search was supplemented with peer-reviewed published articles from the articles’ references, relevant peer-reviewed published articles which were not retrieved by the initial search, unpublished data, and conference proceedings. The process is outlined in Supplementary Fig. 1.

The search identified 363 articles. After filtering through the exclusion criteria (animal research, case reports, comment, editorial, letter, and non-English articles, or articles published outside of the search period), 183 articles were screened and 35 articles were reviewed. In addition, 5 articles were identified as relevant and 1 unpublished dataset was included. In total, 40 studies were included in this review (Supplementary Fig. 1). Within these datasets, the reviewed data included study design, study size, follow-up duration, types and dosing of medications, imaging modalities, and key findings of the articles.

**Supplementary Table 1** Literature search strategy

| **Search number** | **Query** |
| --- | --- |
| 1 | Simultaneous* OR combination* OR concomitant* OR concurrent* OR coexist* OR accompanying OR Coincide* OR coinciding* OR "Co-occur*" OR "Secondary" |
| 2 | "Geographic Atrophy"[Majr] OR "Atrophy" or "Geographic Atrophy" or "Macular atrophy" or "Secondary atrophy" |
| 3 | "Choroidal Neovascularization"[Majr] OR "Choroidal neovascularization*" OR "Choroid neovascularization*" OR "Macular neovascularization*" OR "neovascularization*" OR "Choroidal neovascularisation*" OR "Choroid neovascularisation*" OR "Macular neovascularisation*" OR "neovascularisation*" OR CNV OR MNV |
| 4 | #1 AND #2 AND #3 |
| 5 | "Macular Degeneration"[Majr] OR "Age-related macular degeneration" OR AMD |
| 6 | Definition* |
| 7 | Classification* |
| 8 | Aetiolog* OR etiolog* OR "etiology" [Subheading] |
| 9 | Diagnos* OR "Diagnosis"[Majr] OR "diagnosis" [Subheading] |
| 10 | Imaging OR "Optical Imaging"[Majr] |
| 11 | "Colour photograph*" |
| 12 | Infrared OR "Infra-red" OR "Tomography, Optical"[Majr] |
| 13 | Autofluoresc* OR fundus |
| 14 | "Optical coherence tomography" OR OCT OR OCTA OR "SS-OCT" OR "SD-OCT" OR "Tomography, Optical Coherence"[Majr] |
| 15 | Treatment* OR "therapy" [Subheading] OR "Therapeutics"[Majr] OR therap* |
| 16 | "Anti-vascular endothelial growth factor*" OR "Anti-VEGF*" OR AntiVEGF* |
| 17 | Regimen OR Regimens OR "Clinical Protocols"[Majr] OR "treatment protocol*" OR "clinical protocol*" |
| 18 | "Clinical trial*" OR "clinical study" OR "clinical studies" OR "Clinical Study" [Publication Type] OR "controlled trial*" OR "controlled study" OR "controlled studies" OR randomized OR randomised OR "phase 3" OR "phase III" OR "phase 2" OR "phase II" OR "phase 1" OR "phase I" |
| 19 | Prognos* OR "Prognosis"[Majr] |
| 20 | Outcome* OR "Treatment Outcome"[Majr] OR "Patient Outcome Assessment"[Majr] |
| 21 | #5 OR #6 OR #7 OR #8 OR #9 OR #10 OR #11 OR #12 OR #13 OR #14 OR #15 OR #16 OR #17 OR #18 OR #19 OR #20 |
| 22 | #4 AND #21 |
| 23 | "animals"[MeSH Terms:noexp] NOT ("humans"[MeSH Terms] AND "animals"[MeSH Terms:noexp]) |
| 24 | #22 NOT #23 |
| 25 | "case reports"[Publication Type] OR "comment"[Publication Type] OR "editorial"[Publication Type] OR "letter"[Publication Type] |
| 26 | #24 NOT #25 |
| 27 | #26 AND english[Language] |
| 28 | #27 AND ("2012"[Date - Publication] : "3000"[Date - Publication]) |

**Supplementary Fig. 1** Review process

**
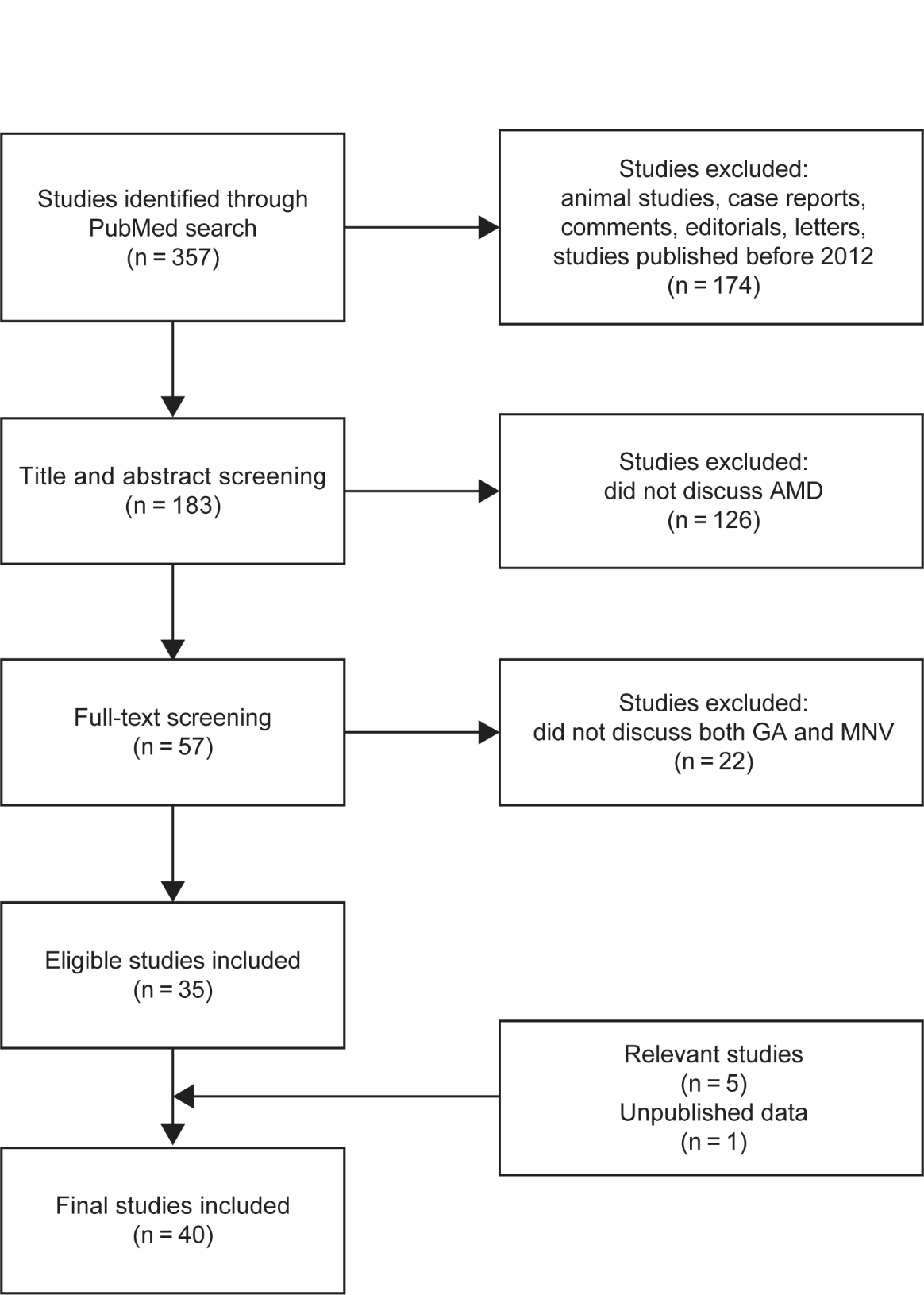
**

*AMD*, age-related macular degeneration; *GA*, geographic atrophy; *MNV*, macular neovascularization
